# Epigenetic regulatory functions of DNA modifications: 5-methylcytosine and beyond

**DOI:** 10.1186/s13072-015-0016-6

**Published:** 2015-07-21

**Authors:** Achim Breiling, Frank Lyko

**Affiliations:** Division of Epigenetics, DKFZ-ZMBH Alliance, German Cancer Research Center, Im Neuenheimer Feld 580, 69120 Heidelberg, Germany

**Keywords:** DNA modification, Modified DNA bases, DNA methylation, Epigenetic marks, Gene regulation, Chromatin, Inheritance, 5-Methylcytosine, 5-Hydroxymethylcytosine, N6-methyladenine, DNA demethylation

## Abstract

The chemical modification of DNA bases plays a key role in epigenetic gene regulation. While much attention has been focused on the classical epigenetic mark, 5-methylcytosine, the field garnered increased interest through the recent discovery of additional modifications. In this review, we focus on the epigenetic regulatory roles of DNA modifications in animals. We present the symmetric modification of 5-methylcytosine on CpG dinucleotide as a key feature, because it permits the inheritance of methylation patterns through DNA replication. However, the distribution patterns of cytosine methylation are not conserved in animals and independent molecular functions will likely be identified. Furthermore, the discovery of enzymes that catalyse the hydroxylation of 5-methylcytosine to 5-hydroxymethylcytosine not only identified an active demethylation pathway, but also a candidate for a new epigenetic mark associated with activated transcription. Most recently, N6-methyladenine was described as an additional eukaryotic DNA modification with epigenetic regulatory potential. Interestingly, this modification is also present in genomes that lack canonical cytosine methylation patterns, suggesting independent functions. This newfound diversity of DNA modifications and their potential for combinatorial interactions indicates that the epigenetic DNA code is substantially more complex than previously thought.

## Background

To establish and maintain cellular identity during development, specific memory mechanisms have evolved that regulate gene expression patterns epigenetically. Once determined, these lineage-specific expression profiles have to be maintained through cell divisions. Active or inactive states of gene expression are defined by specific epigenetic modification patterns that are either accessible to transcription factors and activators, or result in a closed chromatin structure that prevents activated transcription [1–3]. Central to this is the concept of epigenetic marks, specific DNA or chromatin modifications that can be inherited through cell divisions. These marks maintain the epigenetic information and serve as interaction sites for specific binder or reader proteins, which include epigenetic modifier enzymes, repressors, chromatin remodeling complexes and the transcription machinery. The most prominent of these marks is the methylation of the carbon-5 of cytosine (5mC), which is traditionally considered incompatible with activated transcription when present near gene regulatory regions. At these regions, 5mC can modulate the binding of transcription factors [4, 5] or induce the binding of specific 5mC-binding proteins that can lead to the recruitment of co-repressor complexes to methylated target promoters [6].

**Figure 1:**
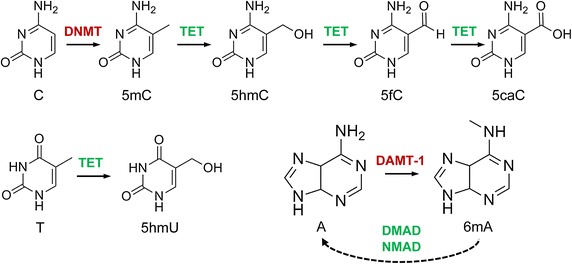
DNA modifications with epigenetic regulatory functions and their interdependencies. Cytosine (C) is methylated to 5-methylcytosine (5mC) by DNA methyltransferases (DNMT) and then further oxidised to 5hmC, 5fC and 5caC by Tet dioxygenases. 5-Hydroxyuracil (5hmU) is produced by Tet-catalysed oxidation of thymine (T). N6-methyladenine (6mA) is likely catalysed by DNA N6 adenine methyltransferases (DAMT-1 in *C. elegans*), even though the biochemical activity of these enzymes remains to be characterized. The Tet-like ALKB enzymes NMAD (N6-methyl adenine demethylase 1) and DMAD (DNA 6mA demethylase) have been shown to be involved in 6mA demethylation in *C. elegans* and in *Drosophila*, respectively, possibly by using a conserved dioxygenase mechanism.

While there is an enormous number of published studies on epigenetic modifications, most of them are correlative in nature. This is exemplified by the increasing use of powerful genome-wide mapping technologies that have revealed numerous associations between changes in epigenetic modification patterns and cell fate transitions [7–9]. However, functional insight remains relatively limited. Furthermore, the field has broadened significantly through the discovery of two additional DNA modifications with epigenetic regulatory functions, 5-hydroxymethylcytosine (5hmC) and N6-methyladenine (6mA), as well as the identification of the corresponding modifying enzymes (Figure 1). Our review aims to illustrate the epigenetic regulatory functions of these DNA modifications, with a predominant focus on animal models. Epigenetic regulation in plants has recently been reviewed elsewhere [10–12].

## 5-Methylcytosine: the fifth base

5-Methylcytosine has been termed the “fifth base” of the human genome. This reflects the relatively high abundance of this modification, as about 4% of the cytosine residues in the human genome have been found to be methylated. However, cytosine methylation levels can differ greatly among animal genomes (see below), and it would therefore be misleading to define the significance of 5mC by its abundance. Rather, the key feature of cytosine methylation is its enrichment or even specificity for “symmetric” CpG dinucleotides [13]. Symmetric methylation means that methylation marks are present on both strands of DNA and that methylation patterns can be faithfully propagated through DNA replication by copying from the parental strand to the unmethylated newly synthesized strand. This methylation maintenance is carried out by the Dnmt1 DNA methyltransferase that has a strong preference for hemimethylated DNA and provides a key paradigm for the stability and heritability of epigenetic information [14]. Dnmt1 is complemented by the Dnmt3 DNA methyltransferases that do not show any selectivity for hemimethylated DNA and have therefore been termed “de novo methyltransferases” [14]. Together, both enzymes catalyze the establishment and maintenance of cytosine DNA methylation patterns during animal development and cell fate specification.

While the overall specificity of animal methylation patterns for CpG dinucleotides has been confirmed in numerous studies, several notable exceptions have also been described. A prominent example is non-CpG methylation in mouse embryonic stem cells (ESCs), which was verified in the first genome-wide methylation analysis of ESCs [15]. While levels of non-CpG methylation are very low in most somatic tissues, extensive postnatal accumulation of this modification has been observed in the mouse and human brain [16–18]. Targeted depletion of Dnmt3a in specific brain regions resulted in significant reduction of non-CpG methylation [18, 19]. In contrast to ESCs where non-CpG methylation seems to correlate with gene expression [15], the modification exhibited an inverse correlation with transcription in neurons, which could partly be explained through the recruitment of the methyl-CpG binding protein 2 (MeCP2) [18, 19]. Context dependent non-CpG methylation might therefore have an impact on specific readers of DNA methylation, thus influencing tissue-specific gene expression.

**Figure 2:**
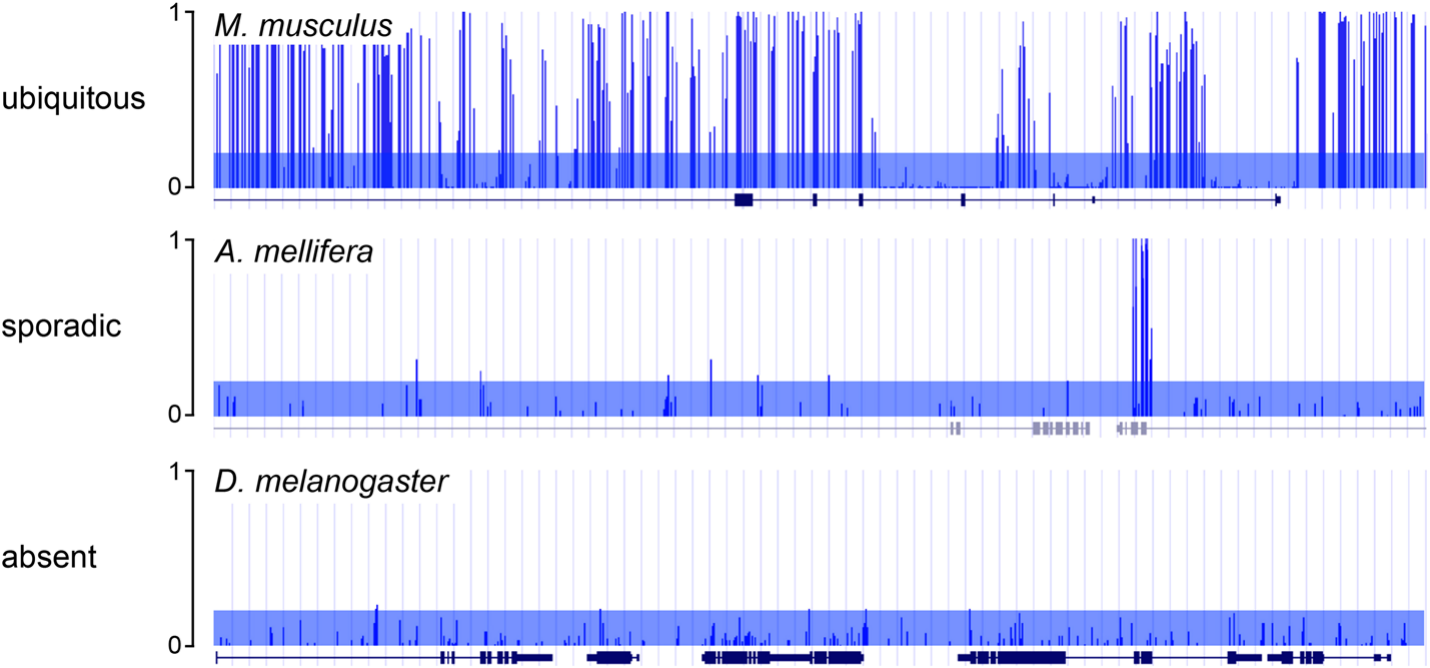
Three major categories of animal methylomes. Ubiquitous, sporadic and absent DNA methylation (5mC) are illustrated with three examples from whole-genome bisulfite sequencing analyses of mouse (*top*), honey bee (*middle*) and *Drosophila* DNA (*bottom*). Methylation ratios for each CpG dinucleotide in a randomly selected 40 kB window are shown. Gene features are indicated below *each panel*. *Transparent blue bars* indicate the range of bisulfite conversion artifacts (methylation ratios below 0.2).

Beyond mammalian methylomes, the comparative analysis of single-base resolution methylation maps has shown a substantial degree of variation between animal species [15, 20, 21]. The available information can be used to define three major categories (Figure 2): the first group is defined by mammalian methylomes and is characterized by pervasive methylation. In the human genome, more than 80% of the CpG dinucleotides are methylated, creating a landscape of ubiquitous methylation, but with local gaps that are often found at active regulatory elements, such as promoters and enhancers (Figure 2). It seems plausible to assume that the default state of these methylomes is “methylated” and that active mechanisms (see below) are required to keep specific regions free of methylation.

The second group is exemplified by the honeybee methylome, that can be defined by only 60,000 CpG-specific methylation marks that are highly enriched in exons [22]. In this case, the default state of the genome appears to be “unmethylated” and the selective targeting of DNA methyltransferases to specific CpGs would be a key step for shaping the methylation landscape (Figure 2). Such sporadic methylation patterns have been described in several animals, particularly in insects. However, the functional significance of sparse methylation remains to be fully understood, which is largely due to the limited potential of the corresponding organisms for genetic manipulation. Importantly, it has been shown that queen-like phenotypes can be enhanced in honeybees following siRNA-mediated knockdown of the Dnmt3 orthologue [23]. While the mechanisms underlying this phenomenon remain to be elucidated, these results strongly suggest a functional role of this enzyme in caste specification, possibly through the modulation of caste-specific methylation patterns.

Finally, several animal genomes have failed to reveal canonical cytosine methylation patterns (Figure 2), which implies that 5mC is not essential for development and cell fate specification of well-known laboratory models such as *S. cerevisiae*, *S. pombe*, *C. elegans* and *D. melanogaster* [24]. The absence of conserved cytosine methylation patterns in these organisms was instrumental for the identification and characterization of other epigenetic mechanisms, including covalent histone modifications and small noncoding RNAs [25–27]. Moreover, it also played an important role in the recent discovery of N6-methyladenine as an epigenetic DNA modification in eukaryotes (see below).

The functional analysis of cytosine methylation has proven to be surprisingly complex and difficult, even in well-characterized mammalian organisms. While knockout models demonstrated a role of Dnmt1 and Dnmt3 in mouse development [28, 29] and in general epigenetic phenomena, such as genomic imprinting [30], X-chromosome inactivation [31] and transposon control [32], the specific function of cytosine methylation in epigenetic gene regulation remains to be fully understood. However, recent integrative studies that combine the targeted disruption of Dnmt genes with genome-wide mapping approaches have provided interesting insight into the functional specificities of individual Dnmts. For example, Dnmt3a-mediated gene body methylation at transcriptionally active genes was shown to be prevalent in postnatal neuronal stem cells and is required for postnatal neurogenesis [33]. In addition, other Dnmts were found to interact with actively transcribed gene bodies, suggesting that gene body methylation promotes transcription [34]. Most recently, Dnmt3b-mediated gene body methylation in mouse ESCs was shown to depend on the presence of histone H3 lysine 36 methylation in the same regions [35]. This represents a novel and unexpected feature of de novo methyltransferases, as it suggests the recruitment of cytosine methyltransferases by the co-transcriptional modification of histones.

In another study, it was shown that human embryonic stem cells lacking both DNMT3A and DNMT3B progressively lose cytosine methylation marks, thus illustrating an imperfect maintenance activity of DNMT1 and a supporting role of DNMT3 enzymes in maintenance methylation [36]. Similar results were obtained with Dnmt-deficient mouse ESCs, which also revealed differential specificities of Dnmt1 and Dnmt3a/b for distinct subclasses of retrotransposons [37]. Further analyses of human ESCs revealed a novel role of DNMT3A in the hypermethylation of genes associated with endoderm differentiation and a rapid, replication-dependent loss of global DNA methylation in DNMT1-deficient cells [36]. It will be important to use similar approaches for the characterization of additional cell types and model systems in order to fully understand the epigenetic regulatory function of 5mC.

## 5-Hydroxymethylcytosine: oxidation creates a new modification

With the discovery of the catalytic dioxygenase activity of Ten eleven translocation (Tet) proteins, novel epigenetic DNA modifications started to emerge [38, 39]. 5-Hydroxymethylcytosine (5hmC, Figure 1) was originally discovered in mammalian DNA in 1972 [40], but its biological significance was investigated only almost 40 years later [41]. Cytosine hydroxymethylation levels are often around 0.1% in mammalian tissues, but can vary greatly [42], with highest values in the brain, where up to 1% of the cytosines can be hydroxymethylated [41]. The three mammalian Tet homologues generate 5hmC from existing 5mC, which they can further process to 5-formylcytosine (5fC) and 5-carboxylcytosine (5caC, Figure 1) [43, 44]. About 30,000 molecules of 5mC, 1,300 of 5hmC, 20 of 5fC, and 3 of 5caC were found per million Cs in mouse embryonic stem cells [44, 45], indicating a very low abundance of 5fC and 5caC. As both modifications are targeted by base excision repair mechanisms mediated by thymine-DNA-glycosylases, they are mainly interpreted as intermediates of an active demethylation pathway via Tet-dependent 5mC oxidation [43, 44].

We are only beginning to understand the functional significance of 5hmC as an epigenetic mark and the specific roles of the three Tet enzymes. Tet1 and Tet2 are highly expressed in mouse ESCs, but their single depletion does not affect pluripotency or development [46–49]. Tet3 homozygous mutant mice develop properly, but die at birth [50], suggesting that Tet3 is also dispensable for embryonic development. ESCs deficient for both Tet1 and Tet2 show insignificant levels of 5hmC, but retain pluripotency. However, the majority of mice lacking both proteins showed developmental defects, which was found to be associated with ectopic hypermethylation [51]. Combined deficiency of all three Tet proteins in ESCs depleted 5hmC completely, but did not affect ESC viability and pluripotency [52–54]. Nevertheless, triple knockout ESCs and embryoid bodies showed impaired differentiation potential, promoter hypermethylation and correlated deregulation of genes implicated in embryonic development and differentiation [52]. In agreement, severe defects in somatic cell reprogramming and mesenchymal-epithelial transition have been described in double and triple Tet knockout mouse embryonic fibroblasts [53].

These data point to a major role of Tet-mediated oxidation in DNA demethylation, most likely by keeping regulatory genomic regions free of 5mC. Particularly important are enhancers, that have been shown to be hypermethylated in Tet-deficient mouse ESCs, resulting in a reduced activity of associated differentiation genes [54, 55]. Tet-dependent oxidation of 5mC as a first step of active demethylation is therefore an early event of enhancer activation [54–56], but might also more generally allow functional interactions with regulatory DNA elements and counteract aberrant spreading of DNA methylation into CpG islands [57].

Nevertheless, 5hmC was also found as a relative stable base at a subset of mammalian promoters, at gene bodies of actively transcribed genes and at poised and active enhancers [58, 59]. 5fC was also mapped to a subset of these 5hmC-marked regions [60–62], suggesting a role as an independent epigenetic mark. Indeed, several “reader” proteins for oxidised 5mC-derivatives have been identified, which might mediate epigenetic regulation [63, 64]. Among these were, in addition to DNA damage- and repair-related proteins, chromatin modifiers and transcriptional regulators like e.g. MBD3, MeCP2, UHRF2 and FOX transcription factors [64–66]. While the functional relevance and specificity of the interactions remains to be fully understood (e.g. many 5hmC interacting proteins also have significant affinities for 5mC) these readers might recruit chromatin regulatory complexes to their targets and support activated transcription.

A role of 5hmC as active mark is supported by mass spectrometric analyses of isotope labelled DNA form mammalian cell culture and mice showing that 5hmC is mostly a stable modification and not a transient intermediate [67]. The high abundance in post-mitotic brain tissues [41, 42] also suggests a direct epigenetic function of 5hmC. Indeed, 5hmC levels increase during neuronal differentiation and a very stable intragenic enrichment of 5hmC was observed at many active neuron-specific genes [66, 68–70]. These findings suggest that 5hmC functions as epigenetic mark in mammalian neuronal development. This is further supported by the observations that the activated human HOXA cluster becomes stably enriched in 5hmC upon retinoic acid stimulated neuronal differentiation [71] and that increased 5hmC levels at neuronal marker genes in Sirtuin-6-deficient mice induce skewed differentiation versus neuroectoderm [72].

While there is evidence for a direct epigenetic function for 5hmC at least in some tissues, a similar role for its oxidation derivatives appears less likely. The levels of 5fC and 5caC have been found to increase at 5fC sites in thymine-DNA-glycosylase-deficient mouse ESCs, suggesting that 5caC sites primarily represent sites of active demethylation [60–62]. It remains possible that, due to the chemical differences between the oxidised 5mC-derivatives, each modification might attract specific readers. However, considering the relatively strong DNA-damage response triggered by 5fC and 5caC (in contrast to 5hmC) and their very low abundances, it seems more likely that these modifications transiently accumulate at the regions of the hydroxymethylome that undergoes demethylation. In contrast, a subset of 5hmC sites appears to be stable and might act as an independent epigenetic mark. Very recently, it has been shown that Tet proteins can also oxidize thymine to 5-hydroxymethyluracil (5hmU, Figure 1) [73]. Tet-dependent 5hmU is present at levels similar to 5caC in mESCs, increases during early ESC differentiation and recruits specific interacting proteins [73], suggesting an epigenetic function for Tet-dependent 5hmU. Nevertheless, 5hmU paired with adenine is a target for the Smug1 DNA glycosylase [74] and might therefore trigger base excision repair mechanisms. Indeed knock down of Smug1 in mESCs led to increased 5hmU levels [73], indicating that 5hmU might also serve to promote active demethylation by recruiting repair factors to Tet targets.

## N6-methyladenine: revival of an old acquaintance

In bacterial genomes 5mC is outshined by a second base modification, N6-methyladenine (6mA, Figure 1). Adenine methylation has been shown to be essential for the viability of several bacteria, as methylation of GATC sequences by the Dam methylase creates specific marks that are important for DNA replication, chromosome segregation, mismatch repair and the regulation of gene expression [75, 76]. However, several older studies also suggested the presence of 6mA in eukaryotic genomes, even though detection was often indirect and modification levels appeared close to the detection limit [76]. Several unicellular eukaryotes, including the green alga *Chlamydomonas reinhardtii*, had consistently shown comparably high levels of DNA adenine methylation [76], which established this organism as an attractive model to investigate 6mA further.

Over the past few years, several powerful technologies were developed to analyze 6mA in RNA, where this modification plays an important regulatory role. When these methods were adapted to characterize the distribution of 6mA in the *Chlamydomonas* genome, some key characteristics of this modification could be defined [77]. For example, the results showed that the algal adenine methylome consists of about 85,000 fully methylated 6mA sites, corresponding to a global adenine methylation level of approximately 0.4%. Methylation was often found in symmetric ApT target sequences, but there was no evidence for symmetric 6mA methylation. The modification was enriched at promoter regions, and particularly in linker regions between adjacent nucleosomes. The authors propose a model in which the DNA 6mA modification either restricts or marks the positions of nucleosomes near transcriptional start sites in *Chlamydomonas*. As such, the presence of 6mA may position nucleosomes to facilitate initiation of transcription. While these findings are highly interesting, they are difficult to generalize because of a highly specific periodic pattern of nucleosome occupancy around transcriptional start sites in *Chlamydomonas*. Furthermore, the *Chlamydomonas* genome has an unusual pattern of 5mC: it is characterised by low levels of CpG methylation but also contains CHG and CHH methylation in gene bodies, which corresponds to known plant methylation patterns [20].

A parallel study also revealed novel details of adenine DNA methylation in *Caenorhabditis elegans* [78]. Similar to *Chlamydomonas*, adenine methylation was found to be variable, and maximum levels were rather low (0.3%). Mapping of 6mA residues by SMRT sequencing revealed that methylation was targeted to GAGG and AGAA consensus sequences, indicating strand-specific adenine methylation. Interestingly, 6mA accumulated in worms deficient for spr-5 (coding for a H3K4me2 demethylase), an important paradigm of trans-generational epigenetic inheritance [78]. Further work led to the identification of a *C. elegans* DNA adenine demethylase (Nmad-1), belonging to the ALKB family of dioxygenases that also contains the Tet proteins. In addition, the authors identified a candidate DNA adenine methyltransferase (Damt-1) related to bacterial 6mA DNA methyltransferases. This enzyme belongs to a highly conserved family of proteins that is characterized by a C-terminal circularly permuted methyltransferase domain fused to a distinctive N-terminal domain [79]. While the biochemical activity of the enzyme remains to be characterized, deletion of Damt-1 suppressed the trans-generational phenotypes of spr-5 mutant worms, suggesting that 6mA might be a transgenerationally inheritable epigenetic mark.

Additional insight into the function of adenine methylation came from a recent analysis in *Drosophila*. Flies represent a particularly interesting model for DNA modifications, because of the longstanding controversial discussions surrounding the cytosine methylation status of the *Drosophila* genome. In addition, the fly genome encodes an unusual DNA methylation machinery, with no canonical Dnmt1/3 homologue, but with a clear Tet homologue. The former is consistent with the reported absence of Dnmt-dependent cytosine methylation patterns in *Drosophila* [24, 80], but the latter seemed to indicate that methylation may have been overlooked so far. By using highly sensitive mass spectrometry approaches, Zhang et al. have now demonstrated the presence of low (0.07%) but significant levels of adenine methylation during the earliest stages of *Drosophila* embryogenesis [81]. Most interestingly, the authors showed 6mA demethylation by the *Drosophila* Tet homologue DMAD in vitro and a specific increase of 6mA levels in the genomic DNA of DMAD mutants suggesting that DMAD is a 6mA-specific enzyme [81]. Furthermore, both deletion and overexpression of DMAD resulted in lethality, thus demonstrating an important developmental function of 6mA in *Drosophila*. One such function could be the regulation of transposons, as 6mA appeared enriched in transposon regions and transposons marked with 6mA were derepressed in DMAD mutants. Taken together, if 6mA will also be found in significant quantities in the genome of other eukaryotes, it might turn out to be an important carrier of epigenetic information, involved in the regulation of gene expression and possibly playing a complementary role to 5mC at certain loci or during specific stages of development.

## Conclusions

**Figure 3:**
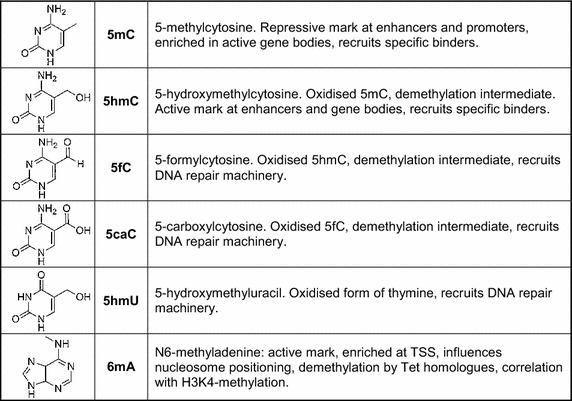
Modified DNA bases and their functions.

Epigenetic DNA modifications generally affect the accessibility of genomic regions for regulatory proteins or protein complexes, for example by preventing interactions or by recruiting specific readers. Consequently, this can influence the chromatin structure and/or directly regulate enhancer and promoter activity or transcriptional processivity. Cytosine methylation is so far the only known symmetric modification with an established maintenance mechanism, which represents a unique feature that currently distinguishes 5mC from all other epigenetic modifications. 5mC has mostly been related to gene repression, in particular at enhancer and promoter regions of genes (Figure 3), but might also play an important role in positively influencing transcription, either by recruiting methylation-specific transcription factors [82, 83] or by a yet to be understood mechanism when present in the body of active genes [35].

Dynamic epigenetic processes also require the active removal of a mark. With the discovery of the enzymatic functions of the Tet proteins, the main enzymes for the removal of DNA methylation were identified. 5hmC and its Tet-dependent oxidation products are demethylation intermediates, but might also have significant roles as independent epigenetic marks (Figure 3). Specific readers for 5hmC, 5fC and 5caC have been identified that function in transcription regulation and chromatin remodeling, mostly promoting the active state. In addition, 5fC, 5caC and 5hmU might primarily function in the recruitment of DNA repair-associated complexes and thus enhance demethylation (Figure 3). Finally, these marks might also directly contribute to gene regulation by triggering “scheduled” DNA repair, which has been suggested to be coupled with activated transcription [84].

The discovery of 6mA in eukaryotes recently identified an additional methylation mark (Figure 3). With *C. elegans* and *D. melanogaster*, two species with negligible 5mC/5hmC levels were shown to contain low, but significant genomic 6mA levels. In both species, this novel modification can be cautiously interpreted as an active epigenetic mark, as data from *C. elegans* suggests a functional interplay with an established active histone mark (H3K4me2) [78], whereas in *Drosophila* mutations in the 6mA-demethylase DMAD (a Tet-homologue) caused increased transposon expression [81]. In both organisms mutations in the 6mA-specific enzymes resulted in significant phenotypes (developmental defects, infertility), suggesting important roles in development. Also in *Chlamydomonas*, 6mA marks actively transcribed genes near the transcriptional start site (TSS).

Future research needs to address the conservation of 6mA and the enzymes that can set and remove this modification. Interestingly, the candidate *C. elegans* 6mA methyltransferase Damt-1 belongs to a widely conserved family of enzymes [78] that also includes a human homologue (METTL4). Nevertheless, reports on 6mA in higher eukaryotes have been sparse and the results were often inconclusive [76]. Highly sensitive mass spectrometry detected less than one molecule of 6mA per million nucleotides in DNA from selected mouse tissues [85], suggesting that 6mA is not a constitutive modification, or is rapidly turned over by demethylation processes. It might be possible to enrich 6mA by depleting the 6mA-demethylase, as shown for *Drosophila* [81]. Furthermore, additional enzymes potentially involved in adenine methylation and demethylation in mammals can be identified using genome editing tools. Finally, the observation that 6mA demethylation in *Drosophila* can be mediated by a Tet-like enzyme [81], raises the fascinating possibility that cytosine and adenine (de)methylation are coordinated. It will be most interesting to investigate the potential interplay between specific DNA modifications and to explore the full complexity of this epigenetic code.

## Abbreviations

5mC: 5-methylcytosine
5hmC: 5-hydroxymethylcytosine
5hmU: 5-hydroxymethyluracil
5caC: 5-carboxylcytosine
5fC: 5-formylcytosine
6mA: N6-methyladenine
ALKB: alpha-ketoglutarate-dependent dioxygenase
CpG: a cytosine followed by a guanine nucleotide
CHG: a cytosine followed by a non-guanine nucleotide followed by a guanine
CHH: a cytosine followed by two non-guanine nucleotides
Dam: deoxyadenosine methylase
Damt: 6mA DNA methyltransferase
Dmad: Drosophila DNA 6mA demethylase
DNA: deoxyribonucleic acid
DNMT: DNA methyltransferase
ESCs: embryonic stem cells
Fox: forkhead box
H3K4me2: dimethylation of lysine 4 at histone H3
MBD: methyl-CpG-binding domain
MECP: methyl-CpG-binding protein
Nmad: N6-methyladenine demethylase
RNA: ribonucleic acid
siRNA: small interfering RNA
SMRT: single molecule real time
Smug: single-strand-selective monofunctional uracil-DNA glycosylase
Spr: suppressor of presenilin
Tet: ten eleven translocation
TSS: transcriptional start site
Uhrf: ubiquitin-like with PHD and ring finger domains

## References

[1] Bird A (2007). Perceptions of epigenetics. Nature.

[2] Kouzarides T (2007). Chromatin modifications and their function. Cell.

[3] Sexton T, Cavalli G (2015). The role of chromosome domains in shaping the functional genome. Cell.

[4] Smith ZD, Meissner A (2013). DNA methylation: roles in mammalian development. Nat Rev Genet.

[5] Baubec T, Schubeler D (2014). Genomic patterns and context specific interpretation of DNA methylation. Curr Opin Genet Dev.

[6] Klose RJ, Bird AP (2006). Genomic DNA methylation: the mark and its mediators. Trends Biochem Sci.

[7] Xie W, Schultz MD, Lister R, Hou Z, Rajagopal N, Ray P (2013). Epigenomic analysis of multilineage differentiation of human embryonic stem cells. Cell.

[8] Gifford CA, Ziller MJ, Gu H, Trapnell C, Donaghey J, Tsankov A (2013). Transcriptional and epigenetic dynamics during specification of human embryonic stem cells. Cell.

[9] Roadmap Epigenomics C, Kundaje A, Meuleman W, Ernst J, Bilenky M, Yen A (2015). Integrative analysis of 111 reference human epigenomes. Nature.

[10] Feng S, Jacobsen SE (2011). Epigenetic modifications in plants: an evolutionary perspective. Curr Opin Plant Biol.

[11] Bemer M, Grossniklaus U (2012). Dynamic regulation of Polycomb group activity during plant development. Curr Opin Plant Biol.

[12] Pikaard CS, Mittelsten Scheid O (2014). Epigenetic regulation in plants. Cold Spring Harb Perspect Biol.

[13] Jones PA (2012). Functions of DNA methylation: islands, start sites, gene bodies and beyond. Nat Rev Genet.

[14] Goll MG, Bestor TH (2005). Eukaryotic cytosine methyltransferases. Annu Rev Biochem.

[15] Lister R, Pelizzola M, Dowen RH, Hawkins RD, Hon G, Tonti-Filippini J (2009). Human DNA methylomes at base resolution show widespread epigenomic differences. Nature.

[16] Xie W, Barr CL, Kim A, Yue F, Lee AY, Eubanks J (2012). Base-resolution analyses of sequence and parent-of-origin dependent DNA methylation in the mouse genome. Cell.

[17] Lister R, Mukamel EA, Nery JR, Urich M, Puddifoot CA, Johnson ND (2013). Global epigenomic reconfiguration during mammalian brain development. Science.

[18] Guo JU, Su Y, Shin JH, Shin J, Li H, Xie B (2014). Distribution, recognition and regulation of non-CpG methylation in the adult mammalian brain. Nat Neurosci.

[19] Gabel HW, Kinde B, Stroud H, Gilbert CS, Harmin DA, Kastan NR (2015). Disruption of DNA-methylation-dependent long gene repression in Rett syndrome. Nature.

[20] Feng S, Cokus SJ, Zhang X, Chen PY, Bostick M, Goll MG (2010). Conservation and divergence of methylation patterning in plants and animals. Proc Natl Acad Sci USA.

[21] Zemach A, McDaniel IE, Silva P, Zilberman D (2010). Genome-wide evolutionary analysis of eukaryotic DNA methylation. Science.

[22] Lyko F, Foret S, Kucharski R, Wolf S, Falckenhayn C, Maleszka R (2010). The honey bee epigenomes: differential methylation of brain DNA in queens and workers. PLoS Biol.

[23] Kucharski R, Maleszka J, Foret S, Maleszka R (2008). Nutritional control of reproductive status in honeybees via DNA methylation. Science.

[24] Raddatz G, Guzzardo PM, Olova N, Fantappie MR, Rampp M, Schaefer M (2013). Dnmt2-dependent methylomes lack defined DNA methylation patterns. Proc Natl Acad Sci USA.

[25] Beisel C, Paro R (2011). Silencing chromatin: comparing modes and mechanisms. Nat Rev Genet.

[26] Lejeune E, Allshire RC (2011). Common ground: small RNA programming and chromatin modifications. Curr Opin Cell Biol.

[27] Reyes-Turcu FE, Grewal SI (2012). Different means, same end-heterochromatin formation by RNAi and RNAi-independent RNA processing factors in fission yeast. Curr Opin Genet Dev.

[28] Li E, Bestor TH, Jaenisch R (1992). Targeted mutation of the DNA methyltransferase gene results in embryonic lethality. Cell.

[29] Okano M, Bell DW, Haber DA, Li E (1999). DNA methyltransferases Dnmt3a and Dnmt3b are essential for de novo methylation and mammalian development. Cell.

[30] Li E, Beard C, Jaenisch R (1993). Role for DNA methylation in genomic imprinting. Nature.

[31] Panning B, Jaenisch R (1996). DNA hypomethylation can activate *Xist* expression and silence X-linked genes. Genes Dev.

[32] Walsh CP, Chaillet JR, Bestor TH (1998). Transcription of IAP endogenous retroviruses is constrained by cytosine methylation. Nat Genet.

[33] Wu H, Coskun V, Tao J, Xie W, Ge W, Yoshikawa K (2010). Dnmt3a-dependent nonpromoter DNA methylation facilitates transcription of neurogenic genes. Science.

[34] Jin B, Ernst J, Tiedemann RL, Xu H, Sureshchandra S, Kellis M (2012). Linking DNA methyltransferases to epigenetic marks and nucleosome structure genome-wide in human tumor cells. Cell Rep.

[35] Baubec T, Colombo DF, Wirbelauer C, Schmidt J, Burger L, Krebs AR (2015). Genomic profiling of DNA methyltransferases reveals a role for DNMT3B in genic methylation. Nature.

[36] Liao J, Karnik R, Gu H, Ziller MJ, Clement K, Tsankov AM (2015). Targeted disruption of DNMT1, DNMT3A and DNMT3B in human embryonic stem cells. Nat Genet.

[37] Li Z, Dai H, Martos SN, Xu B, Gao Y, Li T (2015). Distinct roles of DNMT1-dependent and DNMT1-independent methylation patterns in the genome of mouse embryonic stem cells. Genome Biol.

[38] Tahiliani M, Koh KP, Shen Y, Pastor WA, Bandukwala H, Brudno Y (2009). Conversion of 5-methylcytosine to 5-hydroxymethylcytosine in mammalian DNA by MLL partner TET1. Science.

[39] Ito S, D’Alessio AC, Taranova OV, Hong K, Sowers LC, Zhang Y (2010). Role of Tet proteins in 5mC to 5hmC conversion, ES-cell self-renewal and inner cell mass specification. Nature.

[40] Penn NW, Suwalski R, O’Riley C, Bojanowski K, Yura R (1972). The presence of 5-hydroxymethylcytosine in animal deoxyribonucleic acid. Biochem J.

[41] Kriaucionis S, Heintz N (2009). The nuclear DNA base 5-hydroxymethylcytosine is present in Purkinje neurons and the brain. Science.

[42] Globisch D, Munzel M, Muller M, Michalakis S, Wagner M, Koch S (2010). Tissue distribution of 5-hydroxymethylcytosine and search for active demethylation intermediates. PLoS One.

[43] He YF, Li BZ, Li Z, Liu P, Wang Y, Tang Q (2011). Tet-mediated formation of 5-carboxylcytosine and its excision by TDG in mammalian DNA. Science.

[44] Ito S, Shen L, Dai Q, Wu SC, Collins LB, Swenberg JA (2011). Tet proteins can convert 5-methylcytosine to 5-formylcytosine and 5-carboxylcytosine. Science.

[45] Wu H, D’Alessio AC, Ito S, Wang Z, Cui K, Zhao K (2011). Genome-wide analysis of 5-hydroxymethylcytosine distribution reveals its dual function in transcriptional regulation in mouse embryonic stem cells. Genes Dev.

[46] Dawlaty MM, Ganz K, Powell BE, Hu YC, Markoulaki S, Cheng AW (2011). Tet1 is dispensable for maintaining pluripotency and its loss is compatible with embryonic and postnatal development. Cell Stem Cell.

[47] Koh KP, Yabuuchi A, Rao S, Huang Y, Cunniff K, Nardone J (2011). Tet1 and Tet2 regulate 5-hydroxymethylcytosine production and cell lineage specification in mouse embryonic stem cells. Cell Stem Cell.

[48] Ko M, Bandukwala HS, An J, Lamperti ED, Thompson EC, Hastie R (2011). Ten-Eleven-Translocation 2 (TET2) negatively regulates homeostasis and differentiation of hematopoietic stem cells in mice. Proc Natl Acad Sci USA.

[49] Li Z, Cai X, Cai CL, Wang J, Zhang W, Petersen BE (2011). Deletion of Tet2 in mice leads to dysregulated hematopoietic stem cells and subsequent development of myeloid malignancies. Blood.

[50] Gu TP, Guo F, Yang H, Wu HP, Xu GF, Liu W (2011). The role of Tet3 DNA dioxygenase in epigenetic reprogramming by oocytes. Nature.

[51] Dawlaty MM, Breiling A, Le T, Raddatz G, Barrasa MI, Cheng AW (2013). Combined deficiency of Tet1 and Tet2 causes epigenetic abnormalities but is compatible with postnatal development. Dev Cell.

[52] Dawlaty MM, Breiling A, Le T, Barrasa MI, Raddatz G, Gao Q (2014). Loss of Tet enzymes compromises proper differentiation of embryonic stem cells. Dev Cell.

[53] Hu X, Zhang L, Mao SQ, Li Z, Chen J, Zhang RR (2014). Tet and TDG mediate DNA demethylation essential for mesenchymal-to-epithelial transition in somatic cell reprogramming. Cell Stem Cell.

[54] Lu F, Liu Y, Jiang L, Yamaguchi S, Zhang Y (2014). Role of Tet proteins in enhancer activity and telomere elongation. Genes Dev.

[55] Hon GC, Song CX, Du T, Jin F, Selvaraj S, Lee AY (2014). 5mC oxidation by Tet2 modulates enhancer activity and timing of transcriptome reprogramming during differentiation. Mol Cell.

[56] Serandour AA, Avner S, Oger F, Bizot M, Percevault F, Lucchetti-Miganeh C (2012). Dynamic hydroxymethylation of deoxyribonucleic acid marks differentiation-associated enhancers. Nucleic Acids Res.

[57] Jin C, Lu Y, Jelinek J, Liang S, Estecio MR, Barton MC (2014). TET1 is a maintenance DNA demethylase that prevents methylation spreading in differentiated cells. Nucleic Acids Res.

[58] Pastor WA, Aravind L, Rao A (2013). TETonic shift: biological roles of TET proteins in DNA demethylation and transcription. Nat Rev Mol Cell Biol.

[59] Wu H, Zhang Y (2011). Mechanisms and functions of Tet protein-mediated 5-methylcytosine oxidation. Genes Dev.

[60] Raiber EA, Beraldi D, Ficz G, Burgess HE, Branco MR, Murat P (2012). Genome-wide distribution of 5-formylcytosine in embryonic stem cells is associated with transcription and depends on thymine DNA glycosylase. Genome Biol.

[61] Shen L, Wu H, Diep D, Yamaguchi S, D’Alessio AC, Fung HL (2013). Genome-wide analysis reveals TET- and TDG-dependent 5-methylcytosine oxidation dynamics. Cell.

[62] Song CX, Szulwach KE, Dai Q, Fu Y, Mao SQ, Lin L (2013). Genome-wide profiling of 5-formylcytosine reveals its roles in epigenetic priming. Cell.

[63] Spruijt CG, Gnerlich F, Smits AH, Pfaffeneder T, Jansen PW, Bauer C (2013). Dynamic readers for 5-(hydroxy)methylcytosine and its oxidized derivatives. Cell.

[64] Iurlaro M, Ficz G, Oxley D, Raiber EA, Bachman M, Booth MJ (2013). A screen for hydroxymethylcytosine and formylcytosine binding proteins suggests functions in transcription and chromatin regulation. Genome Biol.

[65] Yildirim O, Li R, Hung JH, Chen PB, Dong X, Ee LS (2011). Mbd3/NURD complex regulates expression of 5-hydroxymethylcytosine marked genes in embryonic stem cells. Cell.

[66] Mellen M, Ayata P, Dewell S, Kriaucionis S, Heintz N (2012). MeCP2 binds to 5hmC enriched within active genes and accessible chromatin in the nervous system. Cell.

[67] Bachman M, Uribe-Lewis S, Yang X, Williams M, Murrell A, Balasubramanian S (2014). 5-Hydroxymethylcytosine is a predominantly stable DNA modification. Nature chemistry.

[68] Song CX, Szulwach KE, Fu Y, Dai Q, Yi C, Li X (2011). Selective chemical labeling reveals the genome-wide distribution of 5-hydroxymethylcytosine. Nat Biotechnol.

[69] Szulwach KE, Li X, Li Y, Song CX, Wu H, Dai Q (2011). 5-hmC-mediated epigenetic dynamics during postnatal neurodevelopment and aging. Nat Neurosci.

[70] Hahn MA, Qiu R, Wu X, Li AX, Zhang H, Wang J (2013). Dynamics of 5-hydroxymethylcytosine and chromatin marks in Mammalian neurogenesis. Cell Rep.

[71] Bocker MT, Tuorto F, Raddatz G, Musch T, Yang FC, Xu M (2012). Hydroxylation of 5-methylcytosine by TET2 maintains the active state of the mammalian HOXA cluster. Nat Commun.

[72] Etchegaray JP, Chavez L, Huang Y, Ross KN, Choi J, Martinez-Pastor B (2015). The histone deacetylase SIRT6 controls embryonic stem cell fate via TET-mediated production of 5-hydroxymethylcytosine. Nat Cell Biol.

[73] Pfaffeneder T, Spada F, Wagner M, Brandmayr C, Laube SK, Eisen D (2014). Tet oxidizes thymine to 5-hydroxymethyluracil in mouse embryonic stem cell DNA. Nat Chem Biol.

[74] Cortellino S, Xu J, Sannai M, Moore R, Caretti E, Cigliano A (2011). Thymine DNA glycosylase is essential for active DNA demethylation by linked deamination-base excision repair. Cell.

[75] Casadesus J, Low D (2006). Epigenetic gene regulation in the bacterial world. Microbiol Mol Biol Rev MMBR.

[76] Ratel D, Ravanat JL, Berger F, Wion D (2006). N6-methyladenine: the other methylated base of DNA. BioEssays.

[77] Fu Y, Luo GZ, Chen K, Deng X, Yu M, Han D (2015). N-Methyldeoxyadenosine marks active transcription start sites in chlamydomonas. Cell.

[78] Greer EL, Blanco MA, Gu L, Sendinc E, Liu J, Aristizabal-Corrales D (2015). DNA Methylation on N-Adenine in C. elegans. Cell.

[79] Iyer LM, Abhiman S, Aravind L (2011). Natural history of eukaryotic DNA methylation systems. Prog Mol Biol Transl Sci.

[80] Takayama S, Dhahbi J, Roberts A, Mao G, Heo SJ, Pachter L (2014). Genome methylation in D. melanogaster is found at specific short motifs and is independent of DNMT2 activity. Genome Res.

[81] Zhang G, Huang H, Liu D, Cheng Y, Liu X, Zhang W (2015). N-methyladenine DNA modification in Drosophila. Cell.

[82] Buck-Koehntop BA, Defossez PA (2013). On how mammalian transcription factors recognize methylated DNA. Epigenetics.

[83] Spruijt CG, Vermeulen M (2014). DNA methylation: old dog, new tricks?. Nat Struct Mol Biol.

[84] Fong YW, Cattoglio C, Tjian R (2013). The intertwined roles of transcription and repair proteins. Mol Cell.

[85] Ratel D, Ravanat JL, Charles MP, Platet N, Breuillaud L, Lunardi J (2006). Undetectable levels of N6-methyl adenine in mouse DNA: cloning and analysis of PRED28, a gene coding for a putative mammalian DNA adenine methyltransferase. FEBS Lett.

